# Advocacy for the human rights of older people in the COVID pandemic and beyond: a call to mental health professionals

**DOI:** 10.1017/S1041610220001076

**Published:** 2020-06-03

**Authors:** Carmelle Peisah, Andrew Byrnes, Israel (Issi) Doron, Michael Dark, Gerard Quinn

**Affiliations:** 1 School of Psychiatry and the Ageing Futures Institute, University of New South Wales (UNSW), Australia; 2 Sydney University, Sydney, Australia; 3 Capacity Australia, Sydney, Australia; 4 Faculty of Law and Ageing Futures Institute, UNSW, Australia; 5 Center for Research and Study of Ageing, Department of Gerontology, University of Haifa, Haifa, Israel; 6 California Advocates for Nursing Home Reform, San Francisco, CA, USA; 7 Raoul Wallenberg Institute, Lund, Sweden; 8 Leeds University, UK

## Introduction

In 2020, COVID-19 hit the earth like a comet. The ground that it seared was already pockmarked by systematic violations of human rights of older people (Doron *et al*., 2004; Mantovani *et al*., 2018) particularly those with mental disorders, due to advanced age, frailty, cognitive impairment, multiple mental and physical comorbidities, and social isolation, the so-called multiple jeopardies for disadvantage (Peisah *et al*., 2011). Ageist and mentalist (i.e. discrimination based on mental illness) assumptions have long-fed unequal treatment, segregation, and discrimination of this group (Quinn *et al*., 2019).

The ethical debate during the pandemic has focused on biological vulnerability and higher fatality of older people (Applegate and Ouslander, 2020; Ruan, 2020), societal responses of distributive justice, and specifically, ventilator allocation (Truog *et al*., 2020). There has been relative silence regarding the myriad other human rights issues at stake. The pandemic has brought to the fore longstanding unaddressed gaps in the actualization of older persons’ human rights, especially those in residential care (Meenan *et al*., 2016).

Health care has been notoriously silent about human rights issues and often demonstrates little awareness of human rights frameworks (Byrnes, 2020; Peisah and Jessop, 2020). Now is the time to speak up and act. This commentary is a call to health professionals, particularly those working in geriatric psychiatry, to embrace human rights frameworks as standards of accountability and advocacy in relation to older persons with mental disorders or dementia, whether living in their own homes or in residential care. From the perspectives of a geriatric psychiatrist and international human rights scholars, we outline existing and emerging human rights frameworks and the effects of COVID on this background human rights landscape. We conclude with practical and tangible strategies for health care professionals working in geriatric psychiatry to promote the equal enjoyment of human rights of older persons.

## The background human rights landscape

The rights of persons with disabilities, given more explicit treatment under international law than the rights of older persons, have much relevance to this context. The lack of equal enjoyment of human rights by people with disability and the need for more specificity in generally worded rights guarantees led to the adoption of the United Nations Convention on the Rights of Persons with Disabilities (CRPD). All States that have ratified the CRPD have binding legal obligations under it including under Article 25.d the obligation to ensure that health care professionals provide health care of the same quality to persons with disabilities as to others on the basis of equality.

The key ideas underpinning the CRPD are equality, autonomy, and independence. Of its 50 Articles, the most salient for geriatric psychiatry are:

(1) Article 12: Equal recognition before the law including the right to equal legal capacity, support in exercising that capacity, expression of will and preferences, and the right to be safeguarded against undue influence and abuse;
(2) Article 14: Liberty and security of the person;
(3) Article 16: Freedom from exploitation, violence, and abuse;
(4) Article 19: Living independently and being included in the community;
(5) Article 22: Respect for privacy;
(6) Article 23: Respect for home and family, and relationships; and
(7) Article 25: Enjoyment of the highest attainable standard of health without discrimination, including respecting specific needs that arise on account of disability. Health care professionals are required to provide care of the same quality provided to others on the basis of free and informed consent, calling for a human rights-based practice of health care based on equity of access, autonomy, and dignity.

Existing rights under CRPD as they apply to aged care have not been adequately implemented, nor are rights under other human rights treaties (Dorn and Apter, 2020). These failures have been driven by ageism, systemic inertia, and failure to recognize the specific situations of older persons (Byrnes, 2020; Doron and Apter, 2020). Advocates for a new UN human rights treaty recognize that while existing human rights treaties including CRPD provide important protections, they do not explicitly engage with issues that are of particular relevance to older persons (Byrnes, 2020; Doron and Apter, 2020; Doron and Ithaka, 2015; Herro and Byrnes, 2020; UN Human Rights Council, 2018).

Furthermore, a large “implementation gap” has existed between articulation of human rights principles in human rights frameworks, policies and legal instruments, and the actual enjoyment of such (Biggs and Haapala, 2013). On the ground in pre-COVID aged care, equity, autonomy, respect for will and preferences, and specific needs conferred by disability remained aspirational. In Australia and elsewhere, COVID-19 has exploded on a background of longstanding neglect of both community and residential care sectors (Royal Commission, 2017). Pre-COVID, social ageism has meant that in the face of resource limitations and recession, service provision in aged and dementia care has suffered most (Biggs and Haapala, 2013).

Steel *et al*. (2019) argued that segregation of people with dementia in residential care facilities, frequently under duress and involving confinement, represented violations in regards to support for independent community living (Article 19), non-discrimination (Article 5), liberty and security of the person (Article 14), equality before the law (Article 12), and accessibility (Article 9). Additionally, quality of end-of-life care in such settings has been variable with much of the shortfall involving key human rights of dignity, autonomy, and equitable access to care (Froggatt *et al*., 2020; PACE, 2019). This has been driven by variable care staff competencies in palliative care due to resource limitations impacting staff education and training (Froggatt *et al*., 2020). Additionally, respect for autonomy and honoring will and preferences at end of life have been unachievable goals in many aged care settings struggling to implement advance care planning (Gilissen *et al*., 2020), despite pushes for this dating back some 25 years.

## The effect of the COVID-19 pandemic

These struggles for actualization of older persons’ basic human rights have come to the fore in the COVID pandemic. At the extreme, the revelations of appalling numbers of deaths in residential facilities (BBC News, 2020) show failures of policy and practice in ensuring human rights to life and health, among others. At the same time, in some countries, deaths in such facilities literally “didn’t count” in mortality figures. The default option of segregating older persons has exposed the heightened vulnerability of congregated settings, where it is intrinsically difficult to secure an adequate standard of health and social distancing (EEG, 2020; Sterling, 2020). It also re-emphasized the fact that despite attempts to transform the institutional landscape of residential care for older persons, it still lacks a true, human rights-based culture (Meenan *et al*., 2016).

In the absence of equitable triage systems for people living in nursing homes with Coronavirus infection, this status has constituted a death sentence for many. Notably, the Office for Civil Rights, US Department of Health and Human Services (DHHS) recently required the State of Pennsylvania to revise its Interim Pennsylvania Crisis Standards of Care for Pandemic Guidelines to ensure that persons with disability are not discriminated against. Removing criteria that automatically deprioritize persons with disability, these guidelines mandate individualized assessments based on the best available, relevant, and objective medical evidence to support triaging decisions and stipulate that no one be denied care based on stereotypes, assessments of quality of life, or judgments about a person’s “worth” based on disability (HHS.gov, 2020).

Numbers of deaths are not all that matters either. In the “tsunami of suffering,” equitable access to quality dying has gone by the wayside (Radbruch *et al*., 2020). Access to essential palliative care and support from loved ones during dying have all been severely curtailed, if not blocked in the face of high demands on health systems. Additionally, the costs in terms of resources, of nuanced support for autonomy and giving voice to will and preferences at the end of life, have also been prohibitive beyond blanket policies of getting people “signed up” as soon as possible with advance care plans. This has culminated in precipitous and sometimes non-competent advance care planning undertaken with older people and their families who have not had opportunities to discuss and reflect on wishes and preferences for end-of-life decisions (Lapid *et al*., 2020). This has been further complicated by impaired cognition and delirium associated with COVID-19 or other incidental illnesses in older people which affect capacity, as well as missed opportunities for supported decision-making in such circumstances (Peisah *et al*., 2013).

In an already compromised setting where shortfalls in equitable access to the highest standard of health existed at the outset, it is not surprising that health care delivery has been inconsistent and sporadic when it occurs, with resources diverted to and focused on the acute hospital sector (EEG, 2020; Lacobucci, 2020). In an under-resourced sector, the costs involved in taking on extra staff, dealing with sickness and providing adequate personal protective equipment (PPE), are likely to be prohibitive of quality care (Lacobucci, 2020). This extends beyond the care setting to shortfalls in care provision for older people with mental illness – particularly those with severe mental illness or dementia living in the community (Killapsy, 2020).

Similar resource limitations have precluded a nuanced approach to enforcing social distancing while maintaining social connection and relationships. The pandemic has necessitated a difficult balance between best practices for infection control in residential facilities and rights and autonomy of the people who live in them. The problem from a policy-making perspective has been that crisis-driven decision-making regarding infection control, when divorced from an understanding of the historical staffing challenges and care practices of these facilities, has put residents at greater risk. Many countries enforced outright prohibitions on visitation by families and friends of residents as an effort to curtail spread (CMS, 2020), although an earlier, more flexible approach involved screening, social distancing, and allowance of visiting on compassionate grounds (WHO, 2020).

One the one hand, the failure to police the flexible approach, and on the other hand, the blanket exclusions have taken their toll. The restrictive prohibition of visitation enforced without any respect for autonomy or consent from older persons themselves or their loved ones has had a disastrous effect on the mental health and well-being of older people who, in segregated settings where loneliness abounds (Freedman and Nicolle, 2020; Jeste *et al*., 2020), depend on visits from family and friends for solace and support. At the same time, physical health depends on infection control. In facilities with long histories of understaffing, health, and safety violations, it was often family members who first identified poor hygiene, lack of feeding and hydration, and improper medication management (Sciacca, 2020). The fault lines in the aged care system have opened up.

It takes extra staff and resourcing to ensure that a spouse can safely visit their partner in a nursing home or even stay with them while they are dying. A blanket rule of no visiting is far more cost effective and keeps numbers of deaths down, but not invariably so. Ironically, it has not been family visitors that have brought the infection into care facilities, but staff. Around the world, despite restrictions on visitors through front doors of facilities, the virus quickly came through the back, carried by health care workers forced by low wages to work at multiple facilities simultaneously with insufficient PPE (Read, 2020). The confluence of these events has taken a terrible toll, leaving residents sick and dying alone – sometimes abandoned altogether by the care facilities in which they lived (ABC7 News, 2020).

These violations go hand in hand with a pre-COVID global propensity for ageism, which has reared its head in some sections of the community. This has been borne out in arguments about who has the better claim to emergency medical treatment, whether the economic and social lockdowns and exit strategies have given excessive weight to interests of older cohorts at the expense of younger cohorts and whether intergenerational justice means that younger cohorts will be bearing an unfair proportion of the very large economic costs of the responses to the pandemic.

## Recommendations for advocacy by health care professionals

Health care professionals are well-placed to support human rights of older people at the coalface. Moreover, it is our experience that framing clinical interventions with reference to human rights sometimes causes policy makers or organizations to stop and take notice. We suggest that when human rights inform medical decision-making, that this be explicitly documented in clinical notes. We highlight potential opportunities in everyday medical decision-making (Kayess, 2020) and clinical practice for advocacy:

1. Reference human rights such as rights to equitable access to health, autonomy, and relationships when planning or writing policies or guidelines for health care services, both hospital and community-based, and care facilities;
2. Highlight the importance of autonomy, free and informed consent, supported decision-making, and respect for will and preferences when delivering treatment. Ensure that human rights-based principles of capacity determination are pursued, including but not limited to: (i) presumption of capacity unless there is evidence to rebut that presumption; (ii) capacity is not diagnosis-bound (e.g. based solely on diagnoses of dementia or schizophrenia) (O’Neill and Peisah, 2019; Peisah, 2017);
3. Rigorous and respectful pursuit of will and preferences with authentic advance care planning that is not driven merely by outcomes. Mining the opportunity for the COVID pandemic to increase death literacy in the population and advance care planning in the future (Lapid, 2020);
4. Understanding human rights violations that underpin changed behaviors in dementia with regard to unmet need, will, and preferences (Articles 12 and 25) (Empowered, 2019) arising from social isolation and unmet intimacy. Raising awareness amongst care staff that the right to social connection (Article 23) is as important, if not more so than daily showering, which some people with dementia find distressing and refuse anyway (Farrell-Miller, 1997);
5. Advocating for the right to autonomy and consent with regard to segregation of older people. This might involve actively supporting a family member to connect with, or visit an older person knowing and understanding the risks, or bringing an older person home to live during the pandemic (EEG, 2020);
6. Explicitly reference human rights when doing assessments and writing medicolegal reports, such as for applications for guardianship or protection/administration;
7. Adopting an anti-ageist culture, climate, and daily behavior based on an awareness of existing stereotypes and prejudices against older persons. Policies should ensure that older persons are not categorized or treated solely through the lens of their chronological age (Ayalon, 2020);
8. Ensure rights of older persons to meaningfully participate not only in personal health care decision-making but also in shaping broader, macro-level health care policies regarding their rights in residential care settings during times of crisis such as the COVID-19;
9. Triage for emergency care should be based on individualized assessment not on diagnosis or place of residence, consistent with the settlement reached by the US DHHS Office for Civil Rights with Pennsylvania (HHS.gov, 2020).

## Conclusion

In the face of the stressors of the pandemic and without an understanding of human rights, it is easy for health professionals to go “off piste” with regards to human rights, careering down a traditional path of best interests. Certainly, best interests and medical beneficence remain important drivers of medical decision-making (Lapid *et al*., 2020). However, an understanding of human rights allows us to pursue other equally important goals of autonomy, connectedness, equal treatment, and dignity. Armed with this understanding, it would be a tragic outcome if health professionals missed opportunities for advocacy for the human rights of older people in their everyday clinical work. Moving toward the future, mental health professionals are perfectly placed to join the debate by the UN 111Open Ended Working Group regarding the possible drafting of a new UN treaty on the rights of older people to pursue the very goals discussed here.
